# IGF-1 Signalling Regulates Mitochondria Dynamics and Turnover through a Conserved GSK-3β–Nrf2–BNIP3 Pathway

**DOI:** 10.3390/cells9010147

**Published:** 2020-01-08

**Authors:** Sarah Riis, Joss B. Murray, Rosemary O’Connor

**Affiliations:** Cell Biology Laboratory, School of Biochemistry and Cell Biology, BioSciences Institute, University College Cork, Cork T12 YT20, Ireland; sarah.riis@ucc.ie (S.R.); joss.murray@ucc.ie (J.B.M.)

**Keywords:** IGF-1, IGF-1R, cancer, NFE2L2/Nrf2, BNIP3, NRF1, HIF-1α, autophagy, mitophagy, cell death

## Abstract

The Insulin-like Growth Factor I (IGF-1) signalling pathway is essential for cell growth and facilitates tumourogenic processes. We recently reported that IGF-1 induces a transcriptional programme for mitochondrial biogenesis, while also inducing expression of the mitophagy receptor BCL2/adenovirus E1B 19 kDa protein-interacting protein 3 (BNIP3), suggesting that IGF-1 has a key mitochondria-protective role in cancer cells. Here, we investigated this further and delineated the signaling pathway for BNIP3 induction. We established that IGF-1 induced BNIP3 expression through a known AKT serine/threonine kinase 1 (AKT)-mediated inhibitory phosphorylation on Glycogen Synthase Kinase-3β (GSK-3β), leading to activation of Nuclear Factor Erythroid 2-related Factor 2 (NFE2L2/Nrf2) and acting through the downstream transcriptional regulators Nuclear Respiratory Factor-1 (NRF1) and Hypoxia-inducible Factor 1 subunit α (HIF-1α). Suppression of IGF-1 signaling, Nrf2 or BNIP3 caused the accumulation of elongated mitochondria and altered the mitochondrial dynamics. IGF-1R null Mouse Embryonic Fibroblasts (MEFs) were impaired in the BNIP3 expression and in the capacity to mount a cell survival response in response to serum deprivation or mitochondrial stress. IGF-1 signalling enhanced the cellular capacity to induce autophagosomal turnover in response to activation of either general autophagy or mitophagy. Overall, we conclude that IGF-1 mediated a mitochondria-protective signal that was coordinated through the cytoprotective transcription factor Nrf2. This pathway coupled mitochondrial biogenesis with BNIP3 induction, and increased the cellular capacity for autophagosome turnover, whilst enhancing survival under conditions of metabolic or mitochondrial stress.

## 1. Introduction

The IGF-1 signalling pathway is essential for cell growth and survival [1], and has the potential to enhance tumourigenesis and cancer progression [2,3]. Anticancer therapies targeting the IGF-1 Receptor (IGF-1R) have overall proved disappointing in clinical trials [4,5]. However, the compelling evidence for IGF actions in cancer and its central function in human biology and ageing mean that it is important to better understand the mechanisms and regulation of this signalling pathway such that it may be successfully modulated or targeted for therapeutic benefit.

Mitochondrial dysfunction is a common feature of cancer cells that may arise due to altered fusion/fission dynamics, oxidative stress, metabolic reprogramming and other mitochondrial changes, as reviewed by Vyas and Haigis [6]. Emerging evidence suggests that dysregulation of mitophagy is common in cancer, and that the accumulation of dysfunctional mitochondria drives cancer progression [7]. The balance between mitochondrial biogenesis and mitophagy requires strict regulation to maintain a sustainable mitochondria population in healthy cells [8]. However, it has been proposed that mitophagy and mitochondrial biogenesis are uncoupled in cancer due to the observed upregulation in mitochondrial biogenesis associated with downregulation of mitophagy [7].

We, among others, have shown that IGF-1 signalling modifies mitochondrial function and fitness, including mitochondrial DNA/RNA maintenance, biogenesis, oxidative phosphorylation and suppression of Reactive Oxygen Species (ROS) production [9,10,11]. IGF-1 induces expression of the highly conserved mitochondrial carrier for the pyrimidine nucleotides Solute Carrier Family 25 (Pyrimidine Nucleotide Carrier 1), member 33 (PNC1/SLC25A33), which is a homologue of the essential yeast carrier RIM2 and is essential for mitochondria DNA and RNA maintenance. Interestingly, suppression of PNC1/SLC25A33 expression in cancer cell lines causes a profound mitochondrial dysfunction, an increase in ROS and an induction of the Epithelial Mesenchymal Transition (EMT) [10]. This observation suggested an important function for IGF-1 signaling in the maintenance of healthy mitochondria and the suppression of ROS-induced EMT in cancer cells. We subsequently reported that IGF-1 promotes mitochondrial biogenesis through up-regulation of the transcriptional co-activators Peroxisome proliferator-activated receptor Gamma Coactivator 1-β (PGC-1β) and PGC-1-related coactivator (PRC), whilst inducing expression of the mitophagy receptor BNIP3 (Bcl-2/adenovirus E1B 19 kDa protein-interacting protein 3) [12].

BNIP3 was originally described as a Bcl-2 homology 3 (BH3)-only member of the Bcl-2 family that can interact with and inhibit pro-survival Bcl-2 proteins to induce apoptosis. It is closely related to Bcl-2/adenovirus E1B 19 KDa protein-interacting protein 3-like (BNIP3L/Nix) with both proteins containing a microtube associated protein 1 light chain 3α (LC3)-interacting regions (LIR) domain that can interact with LC3II on autophagosomes to enable mitophagy [13]. Although they exhibit distinct tissue-specific expression, BNIP3 and BNIP3L are both induced by hypoxia to promote mitophagy that is independent of (PTEN induced kinase 1 (PINK1)/Parkin RBR E3 ubiquitin protein ligase (Parkin). Interestingly, BNIP3 expression has been reported to increase in the early stages of breast cancer but it is suppressed as tumours become hypoxic. BNIP3 inactivation or suppression has been associated with glioma, as well as pancreatic, breast and prostate cancer, with murine cancer models suggesting a tumour-suppressor function [14,15,16]. However, it is still not clear how BNIP3 expression, its induction by IGF-1, or the role of mitophagy in cancer cell metabolism is regulated or integrated with mitochondrial biogenesis and mitochondrial function.

We hypothesized that IGF-1 signaling promotes both mitochondrial biogenesis and mitophagy as an underlying mito-protective feature of all cells (normal and transformed). This highly conserved homeostatic signalling pathway is present in *Caenorhabditis elegans*, where the BNIP3/NIX orthologue DAF-16/FOXO Controlled, germline Tumor affecting (DCT-1) couples the regulation of mitochondrial biogenesis with mitophagy to support a healthy lifespan. The *C. elegans* pathway is controlled by Skinhead 1 SKN-1), which is the orthologue of the transcriptional regulator NFE2L2/Nrf2 [17,18].

Here, we delineated the signaling pathway for IGF-1-mediated BNIP3 induction in cancer cell lines and mouse embryonic fibroblasts MEFs. We found that IGF-1-induced BNIP3 expression requires Nrf2 acting through Hypoxia-inducible Factor 1 subunit α (HIF-1α) and NRF1, and this pathway is essential for mitochondrial morphology and dynamics. Moreover, IGF-1 signalling is essential for cell tolerance to nutrient deprivation and mitochondrial stress. We conclude that IGF-1 signals couple the induction of mitochondrial biogenesis with basal levels of mitochondrial turnover through Nrf2 and BNIP3, thus maintaining mitochondrial homeostasis and facilitating cancer progression.

## 2. Materials and Methods

### 2.1. List of Abbreviations

AKT: AKT serine/threonine kinase 1; BSA: Bovine serum albumin; BNIP3: B-cell lymphoma 2 (Bcl-2)/adenovirus E1B 19 kDa protein-interacting protein 3; CCCP: Carbonyl cyanide 3-chlorophenylhydrazone; CM: Complete/control medium; CQ: Chloroquine; DFP: Deferiprone; Drp1: Dynamin-related protein 1; FCCP: Carbonyl cyanide 4-(trifluoromethoxy)phenylhydrazone; GCLC: Glutamate-cysteine ligase catalytic subunit; GSK-3: Glycogen synthase kinase-3; HO1: Heme oxygenase 1; HIF-1α: Hypoxia-inducible factor 1 subunit α; IGF-1: Insulin-like growth factor 1; IGF-1R: Insulin-like growth factor 1 receptor; KEAP1: Kelch-like ECH associated protein 1; PI3-K: Phosphoinositide 3-kinase; LC3: Microtubule associated protein 1 light chain 3α; NRF1: Nuclear respiratory factor-1; Nrf2/NFE2L2; Nuclear factor erythroid 2-related factor 2; PGC-1β: Peroxisome proliferator-activated receptor gamma coactivator 1-β’; PRC: PGC-1-related coactivator; Parkin/PRKN: Parkin RBR E3 Ubiquitin Protein Ligase; PINK1: PTEN induced kinase 1; PBS: Phosphate-buffered saline; TBS: Tris-buffered saline; p70 S6 kinase: Ribosomal protein S6 kinase, 70 kDa, polypeptide 1; PHB1: Prohibitin 1; p62/SQSTM1: Sequestome 1; TOM20: Translocase of outer mitochondrial membrane 20; mTORC1: Mammalian target of rapamycin complex 1; MFN1: Mitofusin 1; MFN2: Mitofusin 2; SS: Serum starvation; TMRM: Tetramethylrhodamine, methyl ester.

### 2.2. Antibodies

Rabbit anti-phospho-IGF-1R (Y1135/1136, #3024), rabbit anti-IGF-1R (#3027), rabbit anti-phospho-AKT (S473, #4060), rabbit anti-AKT (#2920), rabbit anti-NFE2L2/Nrf2 (#12721), rabbit anti-caspase 3 (#9662), rabbit anti-cleaved caspase 3 (#9661), rabbit anti-phospho-GSK-3β (S9, #9336), rabbit anti-phospo-p70 S6 kinase (T371, #9208) and rabbit anti-p70 S6 kinase (#9202) were all obtained from Cell Signaling Technology (Danvers, MA, USA). Rabbit anti-PHB1 (#PA5-19556) was obtained from Thermo Fisher Scientific (Waltham, MA, USA). Rabbit anti-TOM20 (#sc-11415), mouse anti-TOM20 (#sc-17764), anti-α-tubulin (#sc-23948) and mouse anti-p62 (#sc-28359) were obtained from Santa Cruz Biotechnology (Dallas, TX, USA). Mouse anti-BNIP3 (#ab10433) and mouse Total Human OXPHOS WB antibody cocktail (#ab110411) were obtained from Abcam (Cambridge, UK). Mouse anti-β-actin (#A5441) was obtained from Sigma-Aldrich (St. Louis, MO, USA). Mouse anti-GSK-3 (#610202) was obtained from BD Biosciences (Franklin Lakes, NJ, USA) and Rabbit anti-HIF-1α (#A300-286A) was obtained from Bethyl Laboratories Inc. (Montgomery, TX, USA). Rabbit anti-PINK1 (#BC-100-494) was obtained from Novus Biologicals (Littleton, CO, USA).

Of note, BNIP3 is known to undergo post translational modification, including phosphorylations that can affect the migratory pattern, so a series of bands around 30–35 kDa can be seen apart from the two dominant bands representing the monomer at ≈20–25kDa and the dimer ≈55–60 kDa, although the profile varies slightly depending on the cell line [19,20]. All bands were eliminated via suppression of BNIP3 with siRNA, except a band at ≈32 kDa and a faint band at ≈45 kDa that were concluded to be unspecific (see Supplementary Materials Figure S1A). For the quantification of protein from human cancer cell lysates, densitometry was performed measuring the bottom monomer band only, which was consistently detected with different antibody batches, and was specifically altered by the indicated culture conditions. For the MEFs, all bands in the 25–30 kDa range were included for densitometry due to the different antibody detection profile observed in these cells.

### 2.3. Cell Lines and Cell Culture

MCF-7, DU145 and U2OS cells were all obtained from ATCC (Old Town Manassas, VA, USA). MCF-7 breast cancer cells, U2OS osteosarcoma cells and R− and R+ cells (mouse embryonic fibroblast cell lines (MEFs) derived from IGF-1R knock-out mice [21] were all cultured in Dulbecco’s Modified Eagle Medium (DMEM) (#D6429, Sigma) supplemented with 10% heat-inactivated fetal bovine serum, 10 mM l-glutamine and 5 mg/mL penicillin/streptomycin DU145 prostate carcinoma cells were cultured in RPMI-1640 medium (RPMI) (#R8758, Sigma)with the same additions. Prior to experiments using IGF-1 stimulation or chemical inhibitors, cells were cultured for 16 h and allowed to reach approximately 70% confluence. For serum starvation, cells were washed twice with PBS and then cultured in serum-free media for 4 h prior to the addition of IGF-1 (Peprotech #100-11, NJ, USA) at 10 ng/mL for the indicated periods of time.

The PI3-K inhibitor LY294002 (#440204, Merck) at 20 μM was added to cell cultures 30 min prior to the addition of IGF-1. The GSK-3 inhibitor SB415286 (#S3567, Sigma)at 30 μM, the GSK-3 inhibitor LiCl at 10 mM, the iron chelator deferiprone (DFP, #379409, Sigma) at 1 mM or the mitochondrial uncouplers carbonyl cyanide 3-chlorophenylhydrazone (CCCP, #C2759, Sigma) at 10 μM or carbonyl cyanide 4-(trifluoromethoxy)phenylhydrazone (FCCP, #C2920, Sigma), also at 10 μM, were added to cell cultures for the indicated time periods. The lysosomal inhibitor chloroquine (#14774, Sigma) at 50 μM was added for the last 4 h of cell culture in the indicated experiments. LY294002, SB415286, CCCP and FCCP were all dissolved in dimethyl sulfoxide (DMSO), and therefore DMSO alone was added as a vehicle control at the equivalent volume to the control samples.

### 2.4. SiRNA Transfection

SiRNAs targeting BNIP3 (L-004636-00-0005), NFE2L2/Nrf2 (L-003755-00-0005) and NRF1 (L-017924-00-0005) were purchased from Dharmacon (Lafayette, CO, USA). As a negative control, Silencer Negative Control 1 (AM4611) was obtained from Thermo Fisher Scientific (Waltham, MA, USA). siRNA transfections (20 nM) were performed using Lipofectamine RNAiMAX from Thermo Fisher Scientific per manufacturer’s instructions.

### 2.5. Plasmid Transfection

Transfection with DNA plasmids was done with Lipofectamine (#18324-012) for MCF-7 cells or with Lipofectamine 2000 (#11668-019) for DU145. Both were purchased from Thermo Fisher Scientific. The pcDNA3-HA2-KEAP1 plasmid was a kind gift from Yue Xiong (Addgene plasmid #21556; http://n2t.net/addgene:21556; RRID: Addgene_21556).

### 2.6. Immunofluorescence

MCF-7 or DU145 cells (200,000/well) or MEFs (150,000 cells/well) were seeded onto sterile glass coverslips in six-well plates and cultured as described in the Figure 4A,C,E legends prior to fixation for 30 min (MCF-7) or 1 h (DU145 and MEFs) with 4% paraformaldehyde in PBS, followed by quenching with 50 mM NaCl for 15 min and washing with PBS. The cells were then permeabilised with 0.1% Triton-X in PBS for 5 min, followed by a wash with PBS. To prevent non-specific interactions with secondary antibodies, cells were incubated with a blocking buffer containing 5% donkey serum in PBS. The coverslips were incubated with primary antibodies overnight at 4 °C. The anti-TOM20 antibody was used at a 1:500 dilution.

Secondary antibodies (Alexa 488-conjugated, #711-545-152 from Jackson Immuno Research (West Grove, PA, USA)) were applied (prepared per manufacturers instruction and used at 1:200 dilution) for 1 h at room temperature. Images were obtained using a Nikon Eclipse E600 microscope (Micron Optical, Wexford, Ireland) equipped with a SPOT digital camera.

### 2.7. Cell Lysis, SDS-PAGE and Western Blotting

Cells were lysed with RIPA lysis buffer (50 mM Tris, 150 mM NaCl, 0.1% SDS, 0.5% sodium deoxycholate and 1% NonidetP-40 (pH 7.4)) for 20 min on ice. SDS-PAGE was performed on 10 or 15% polyacrylamide gels depending on the size of the proteins analyzed. Generally, protein samples were loaded in the range of 20–80 μg depending on the cell line and the sensitivity of the antibodies used for detection. Proteins were transferred to a nitrocellulose membrane using a Bio-Rad Mini Trans-Blot electrophoretic transfer cell (Web Scientific, Radway Green, UK). Membranes were blocked with 5% non-fat dried milk or BSA in TBS containing 0.05% Tween 20 for 1 h at room temperature, incubated with primary antibodies overnight at 4 °C and subsequently with IRdye700- or IRdye800-conjugated secondary antibodies (LI-COR Biosciences, Cambridge, UK) for 1 h at room temperature. An Odyssey IR scanner system was used for protein detection (LI-COR Biosciences, Cambridge, UK). Molecular masses are indicated on all blots in kilodaltons.

### 2.8. Subcellular Fractionation

Cellular fractionation was performed to obtain cytosolic and nuclear fractions following the protocol published by Lau et al. [22]. The only amendments were that the pellet was washed three times with “buffer A” containing no Triton X-100 rather than once, and the nuclear lysis was performed for 1 h rather than 30 min. Protein concentration was assessed using a Bradford assay, and equal amounts of all samples were loaded onto 10% polyacrylamide gels for SDS-PAGE.

### 2.9. Quantitative Real-Time PCR

RNA extraction was carried out per the manufacturer’s instructions using the Pure Link RNA Mini Kit (Thermo Fisher Scientific, #12183025). The Quanti Tect Reverse Transcription Kit (Qiagen, Hilden, Germany, #205313) was used for cDNA synthesis with 500 ng–1 μg of RNA. Two negative controls were included for each cDNA synthesis, either containing no RNA or no reverse transcriptase to test for contamination and the appropriate elimination of genomic DNA. The FastStart Essential DNA Green Master Kit (Roche Diagnostics, Risch-Rotkreuz, Switzerland, #6402712001) was used for RT-qPCR per the manufacturer’s instructions using a LightCycler96 Instrument (Roche Diagnostics.Ubiquitin C (UBC) was used to normalize gene expression levels for cancer cell lines and β-actin was used for MEFs. For RT-qPCR data analysis, Ct values <30 were considered positive. ΔCt values were calculated via normalisation to the housekeeping gene. ΔΔCt values were calculated as the difference between the control sample and the sample of interest. Finally, the relative fold change was calculated using the 2^ΔΔCT^ method. Gene expression levels were then normalised to the control samples and presented as a relative fold change with the control sample set to a value of 1.

For all RT-qPCRs, the data were derived from three independent experiments (biological repeats) as mean ± SEM. All of the primers used for RT-qPCR are listed in Supplementary Materials Table S1.

### 2.10. Flow Cytometry

Tetramethylrhodamine, methyl ester (TMRM, Thermo Fisher Scientific #T668) dye was used to assess mitochondria membrane potential using flow cytometry because it accumulates in polarized/active mitochondria. R− and R+ cells were seeded at 175,000 cells per well in six-well plates in triplicate wells per condition: TMRM was used at 500 nM and FCCP at 20 μM was added to samples as a positive control for 10 min prior to the addition of TMRM. Cells were incubated in the dark at 37 °C for 20 min, then washed with PBS and detached using accutase (Sigma, #A6964). Harvested cells were centrifuged at 1000 rpm for 5 min, resuspended in PBS and were immediately analysed using a FACSCalibur (BD Bioscences, San Jose, CA, USA) and the Cellquest Pro software (BD Biosciences). The fluorescence intensity was measured in the FL2 channel for 10,000 events per sample. For each sample, the geometric mean was measured and the average was calculated.

### 2.11. Analysis of Transcription Factor Binding Sites in the BNIP3 Promoter Region

The BNIP3 promoter sequence 1000 base pairs upstream of the transcription start site was identified using the Eukaryotic Promoter Database [23] (https://epd.epfl.ch//index.php), and this was overlaid on the BNIP3 sequence in the University of California Santa Cruz (UCSC) Genome Browser [24] (https://genome.ucsc.edu/). Applying the OReGanno tool [25], the promoter was subsequently analysed for potential transcription factor binding sites. The JASPAR scan analysis function [26] was used to predict the binding of NRF1 (matrix ID MA0506.1), HIF-1α (MA1106.1) and Nrf2 (MA0150.1). The software generates a score and a relative score based on the similarity between the probed sequence and the transcription factor consensus sequence.

## 3. Results

### 3.1. Nrf2 Is Required for IGF-1-Mediated Induction of the Mitophagy Receptor BNIP3

Previously we showed that IGF-1 induces the expression of the transcription coactivators PGC-1β and PRC that mediate a mitochondrial biogenesis programme. IGF-1 also induces the expression of the mitophagy receptor BNIP3 at the mRNA and protein level [12]. Since mitochondrial biogenesis and mitophagy may be coupled, and since Nrf2 promotes the transcription of the BNIP3/NIX orthologue DCT-1 in *C. elegans* [18], we hypothesized that Nrf2 activity may be important for the IGF-1-mediated induction of BNIP3 in mammalian cells.

**Figure 1:**
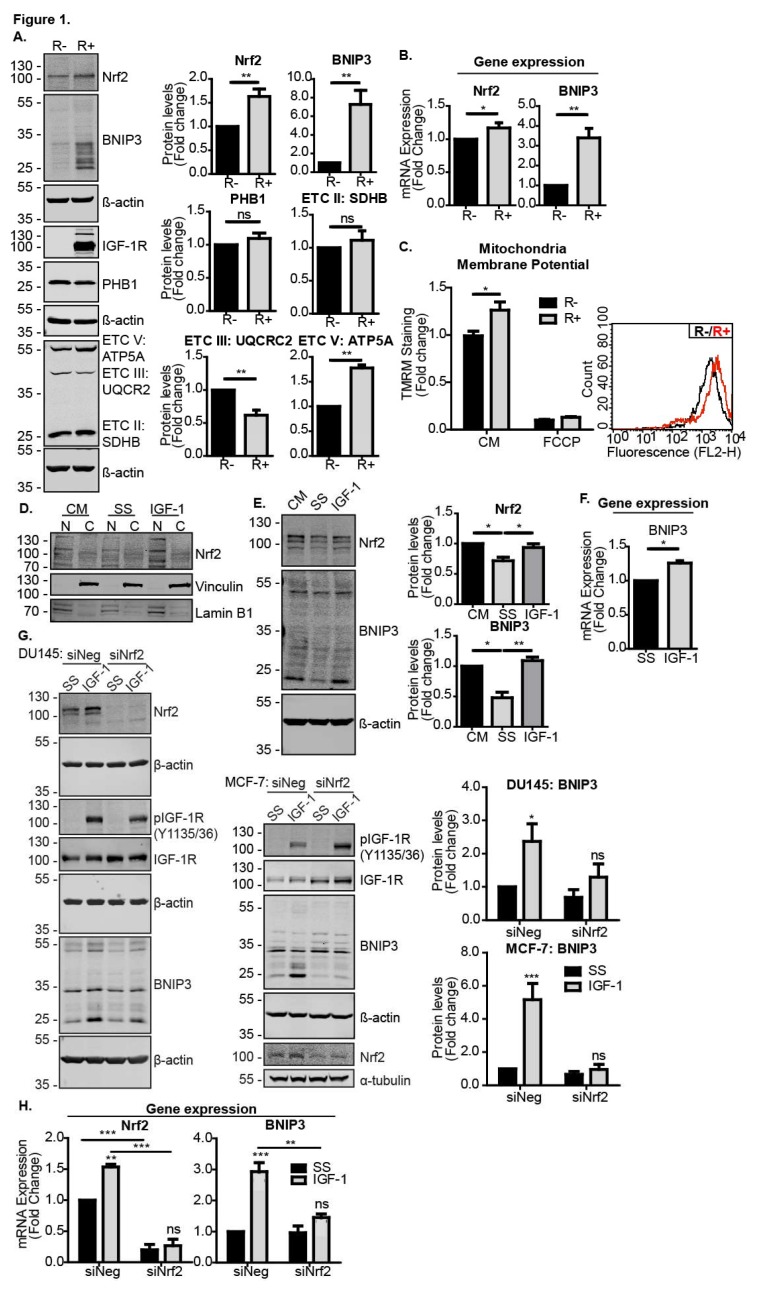
IGF-1-induced BNIP3 expression is Nrf2-dependent. (**A**) Western blot showing levels of Nrf2, BNIP3, PHB1 and OXPHOS components in R− and R+ cells in the control medium (CM). (**B**) Gene expression levels of Nrf2 and BNIP3 in R− and R+ cells in the CM measured using RT-qPCR. (**C**) Mitochondrial membrane potential measured using flow cytometry in R− and R+ cells using TMRM dye. The average geometric means are shown as a relative fold change compared to R− set to a value of 1 for the first biological repeat. FCCP positive controls were included. A representative graph illustrating the fluorescence intensity of R+ cells (red) overlaid onto R− cells (black) is also shown. (**D**) Western blot showing Nrf2 levels in nuclear (N) and cytosolic (C) fractions of MCF-7 cells maintained in a control medium or serum starved for 4 h followed by 4 h of IGF-1 stimulation (10 ng/mL). (**E**) Western blot showing Nrf2 and BNIP3 levels in DU145 cells. The cells were kept in the CM or serum-starved for 4 h prior to the addition of IGF-1 (10 ng/mL) for 20 h. (**F**) Gene expression levels of BNIP3 in U2OS cells measured using RT-qPCR. The cells were serum-starved for 4 h prior to stimulation with IGF-1 (10 ng/mL) for 20 h. (**G**) MCF-7 and DU145 cells were transfected with siNeg or siNrf2. At 48 h post transfection, the cells were serum-starved for 4 h prior to the addition of IGF-1 (10 ng/mL) for 20 h. (**H**) Gene expression levels of Nrf2 and BNIP3 in MCF-7 cells transfected with siNeg or siNrf2 measured using RT-qPCR. At 48 h post transfection, the cells were serum-starved for 4 h prior to addition of IGF-1 at 10 ng/mL for 20 h prior to RNA extraction. In all panels, the data were derived from three independent experiments. For Western blots, protein levels were normalised to β-actin and presented as a fold change relative to the control sample set to a value of 1. For RT-qPCR, gene expression levels were normalised to the housekeeping gene UBC (human cell lines) or β-actin (MEFs) and presented as a fold change relative to control conditions set at a value of 1. Statistical analysis was performed using the Student’s *t*-test (**A**–**C**,**F**), one-way ANOVA (E) or two-way ANOVA (**G**,**H**) (*: *p* < 0.05, **: *p* < 0.01, ***: *p* < 0.001). ATP5A: ATP Synthase F1 Subunit α, ETC V: Electron transport chain, SDHB: Succinate dehydrogenase complex iron sulfur subunit B, UQCR2: Ubiquinol-cytochrome C reductase core protein 2.

To test this, we first compared Nrf2 and BNIP3 levels in IGF-1R KO MEFs (R− cells) and R− cells stably reconstituted with the IGF-1R (R+ cells). As can be seen in Figure 1A,B, R− cells expressed significantly lower levels of both Nrf2 and BNIP3 protein and mRNA than R+ cells, suggesting a requirement for IGF-1 signalling in the expression of these proteins. Expression levels of the mitochondrial marker prohibitin 1 (PHB1) were similar in the two cell lines, indicating that the difference observed in the BNIP3 expression was a significant difference in mitochondrial mass (Figure 1A). Interestingly, expression levels of electron transport chain components were different in R− and R+ cells, with R+ cells exhibiting lower levels of complex III, but higher levels of complex V/ATP synthase, suggesting an altered capacity for oxidative phosphorylation in R+ cells. This was associated with a slightly higher mitochondria membrane potential in R+ cells compared to R− cells, as measured using flow cytometry analysis of cells stained with the TMRM dye (Figure 1C). Thus, the data suggest that IGF-1 was important for the regulation of mitochondria function and potentially turnover.

IGF-1-mediated induction of BNIP3 expression was observed in all cancer cell lines tested, including MCF-7 cells [12]. Here, we found that IGF-1 stimulation also increased the amount of Nrf2 present in the nuclear fraction of MCF-7 cells, which was likely associated with Nrf2 acting as a transcription factor (Figure 1D). We also found that IGF-1 induced Nrf2 and BNIP3 in DU145 cells, where serum starvation reduced the levels of both Nrf2 and BNIP3, and subsequent IGF-1 stimulation induced the expression of both proteins (Figure 1E). The human osteosarcoma cell line U2OS also exhibited an increase in BNIP3 expression in response to IGF-1 (Figure 1F). To test whether Nrf2 was required for the IGF-1-mediated induction of BNIP3, we suppressed Nrf2 with siRNA in MCF-7 and DU145 cells, and observed that this significantly impaired the BNIP3 induction by IGF-1 (Figure 1G). A similar result was observed with BNIP3 transcription because although IGF-1 induced the expression of both Nrf2 and BNIP3 mRNA in MCF-7 cells transfected with a control siRNA, Nrf2 suppression resulted in a loss of IGF-1-induced BNIP3 induction (Figure 1H).

These results demonstrate that Nrf2 was required for the induction of BNIP3 in response to IGF-1.

### 3.2. IGF-1 Induced BNIP3 through an Inhibitory Phosphorylation of GSK-3β

**Figure 2:**
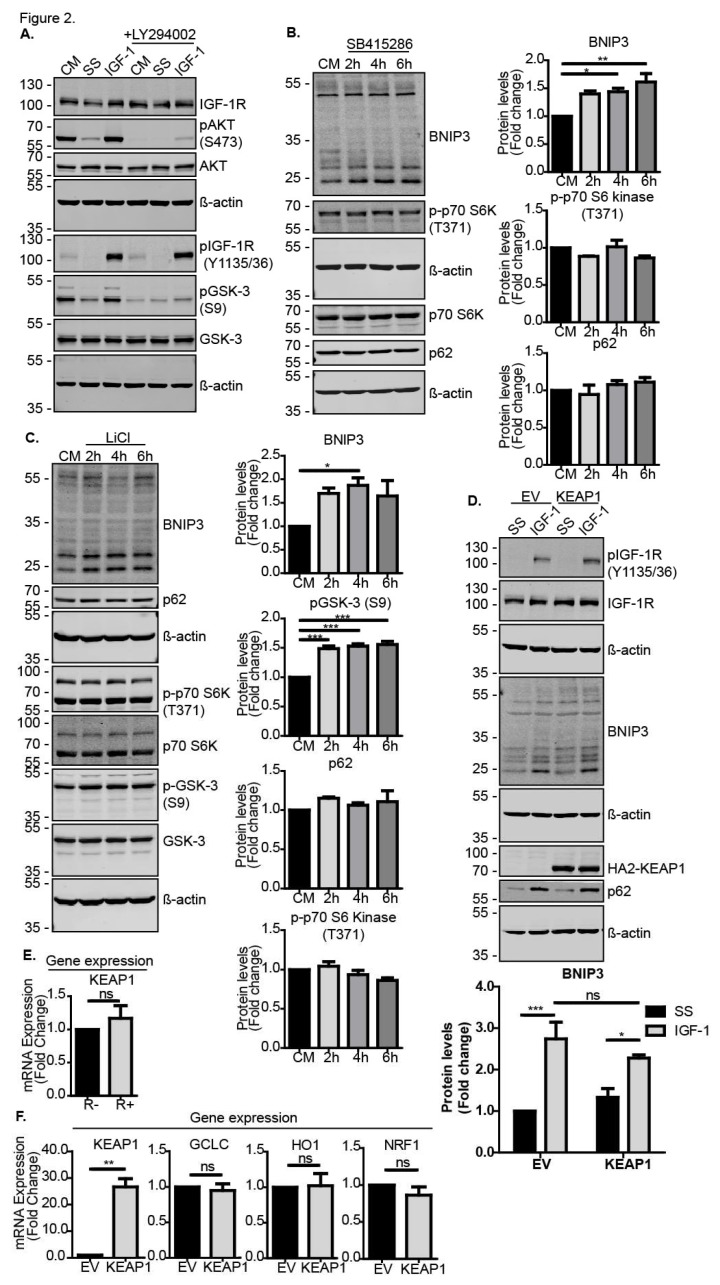
IGF-1-induces BNIP3 protein expression through inhibition of GSK-3. (**A**) Western blot of MCF-7 cells showing levels of AKT phosphorylated on S473 and GSK-3 phosphorylated on S9. Cells were serum-starved for 4 h prior to stimulation with IGF-1 (10 ng/mL) for 4 h in the absence or presence of the AKT inhibitor LY294002 (20 μM) that was added 30 min prior to stimulation. (**B**) Western blot showing levels of Nrf2, BNIP3, p62 and p-p70 S6 kinase (T371) in DU145 cells. Cells were cultured in CM in the presence or absence of 30 μM SB415286 for 2, 4 or 6 h. (**C**) Western blot showing levels of BNIP3, pGSK-3β (S9), p62 and p-p70 S6 kinase in MCF-7 cells. Cells were cultured in the CM in the absence or presence of 10 mM LiCl for 2, 4 or 6 h. (**D**) Western blot of DU145 cells transiently transfected with pcDNA3 empty vector (EV or pcDNA3-HA2-KEAP1. The cells were serum-starved for 4 h prior to stimulation with IGF-1 (10 ng/mL) for 20 h. (**E**) Keap1 expression levels in R− and R+ cells in control media measured using RT-qPCR. (**F**) mRNA expression levels of KEAP1 and Nrf2 target genes, GCLC, HO1 and NRF1 in DU145 cells transfected with pcDNA3.1 empty vector or pcDNA3.1-HA2-KEAP1 for 48 h and cultured in CM. mRNA was measured using RT-qPCR. In all panels, the data presented were derived from three independent experiments. For Western blots, the protein levels were normalised to β-actin and presented as fold-change relative to the control sample set to a value of 1. For RT-qPCR, the gene expression levels were normalised to the housekeeping gene UBC (human cell lines) or β-actin (MEFs) and presented as fold-change relative to control conditions set at a value of 1. Statistical analysis was performed using one-way ANOVA (**B**,**C**), two-way ANOVA (**D**) and Student’s *t*-test (**E**,**F**) (*: *p* < 0.05, **: *p* < 0.01, ***: *p* < 0.001).

We next investigated how the IGF-1 signaling pathway intersected with the induction of Nrf2 expression and activity. Nrf2 protein stability, and thereby its activity, is tightly regulated in cells through inhibitory cytoplasmic protein complexes that may include KEAP1 or GSK-3β [27,28,29], resulting in the degradation of Nrf2. The IGF-1-stimulated activation of AKT via PI3-K can mediate an inhibitory phosphorylation on serine 9 (S9) on GSK-3β [30]; therefore, we next tested whether IGF-1 activated Nrf2 by inhibiting GSK3β-mediated Nrf2 degradation. As expected, IGF-1 enhanced S9 phosphorylation on GSK-3β in MCF-7 cells in a PI3-K-dependent manner because PI3-K inhibition with LY294002 impaired this IGF-1-mediated phosphorylation on S9 (Figure 2A). Moreover, the pharmacological inhibition of GSK-3 with SB415286 in the control medium resulted in higher BNIP3 protein levels in DU145 cells (Figure 2B) and MCF-7 cells (Supplementary Materials Figure S1B), although the induction appeared to be transient in MCF-7 cells. Importantly, SB415286 did not alter the activity of mTORC1, as indicated by the maintenance of p-p70 S6 kinase (T371) levels or cause a significant reduction in p62/sequestome 1 levels (Figure 2B), indicating that general autophagy was unaffected. Similarly, MCF-7 cells (Figure 2C) and DU145 (Supplementary Materials Figure 1C) exposed to LiCl, a known inhibitor of GSK-3β and inducer of Nrf2 [31,32], exhibited increased BNIP3 expression without an associated induction of general autophagy. Thus, these data show that the inhibition of GSK-3β may selectively activate BNIP3 and potentially induce BNIP3-mediated mitophagy.

We also tested whether activity of the Nrf2 negative regulator KEAP1 was implicated in IGF-1-mediated BNIP3 induction. Ectopic expression of KEAP1 in either MCF-7 (Figure 2D) or DU145 (Supplementary Materials Figure S1E) cells did not significantly alter the induction of BNIP3, indicating that the IGF-1 effects on Nrf2 activity were independent of KEAP1. It is possible that the IGF-1-induced accumulation of p62 that occurs due to autophagy inhibition [33], which is evident in in Figure 2D, could have impaired KEAP1 inhibition of Nrf2 because p62 competes with KEAP1 for binding to Nrf2 [34]. Such effects would indirectly enhance Nrf2 stabilization. Furthermore, there was no significant difference in the expression levels of KEAP1 between R− and R+ cells (Figure 2E), and levels of the known Nrf2 target genes GCLC, HO1 and NRF1 were not significantly affected by KEAP1 overexpression in DU145 cells cultured in control medium (Figure 2F) or in MCF-7 cells (Supplementary Materials Figure S1D), except for a slight reduction in GCLC. Taken together, these data indicate that IGF-1 signaling induces Nrf2 expression and protein stabilization through the PI3-K/AKT-mediated phosphorylation of GSK-3β on S9, which prevents GSK-3-mediated Nrf2 degradation.

### 3.3. Nrf2 Induced BNIP3 Expression through HIF-1α and NRF1

**Figure 3:**
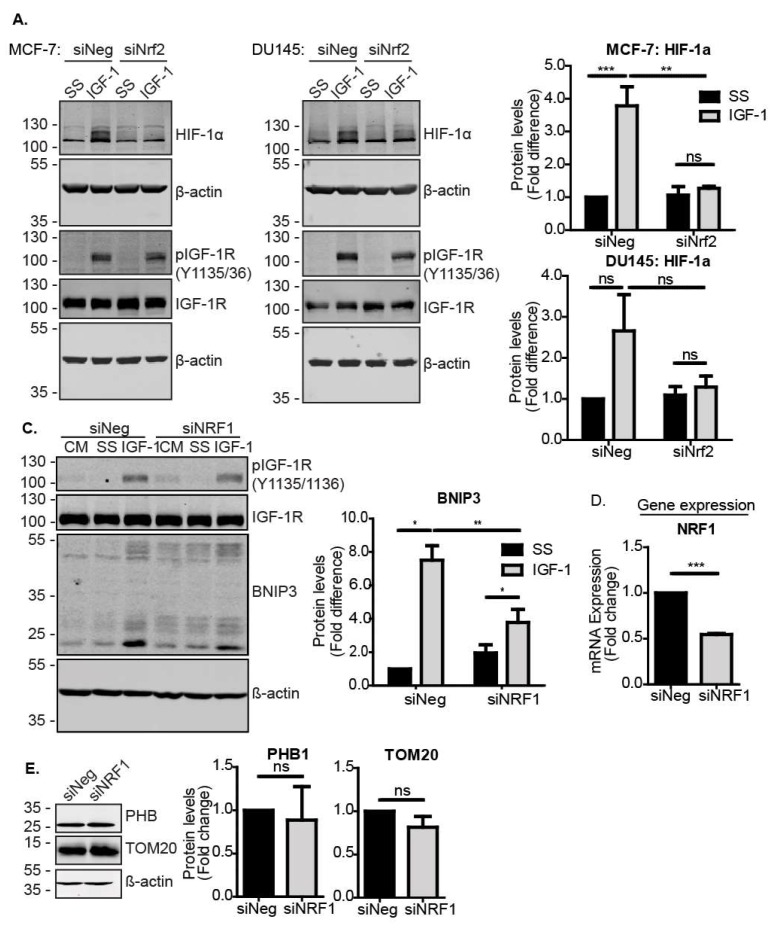
Nrf2 induces BNIP3 through HIF-1α and NRF1. (**A**) Western blot showing levels of HIF-1α and IGF-1R in MCF-7 and DU145 cells transfected with siNeg or siNrf2. At 48 h post transfection, cells were serum-starved for 4 h prior to stimulation with IGF-1 (10 ng/mL) for 20 h. (**B**) Western blot showing the BNIP3 expression MCF-7 cells transfected with siNeg or siNRF1. At 48 h post transfection, cells were serum-starved for 4 h prior to stimulation with IGF-1 (10 ng/mL) for 20 h. (**C**) Expression levels of NRF1 in cells transfected with siNeg or siNRF1 for 72 h measured using RT-qPCR. (**D**) Western blot showing levels of mitochondrial markers PHB1 and TOM20 in MCF-7 cells transfected with siNeg or siNRF1 for 72 h. The cells were cultured in the CM and analysed at 72 h post transfection. In all panels, the data presented were derived from three independent experiments. For Western blots, protein levels were normalised to β-actin and presented as a fold change relative to the control sample set to a value of 1. For RT-qPCR, gene expression levels were normalised to the housekeeping gene UBC (human cell lines) or β-actin (MEFs) and presented as a fold change relative to control conditions set at a value of 1. Statistical analysis was performed using two-way ANOVA (**A**,**C**) and Student’s *t*-test (**D**,**E**) (*: *p* < 0.05, **: *p* < 0.01, ***: *p* < 0.001).

To further investigate how Nrf2 mediates the induction of BNIP3 in response to IGF-1, we asked whether Nrf2 directly induces BNIP3 transcription, or whether other downstream transcription factors are required. For example, it was previously reported that HIF-1α induces BNIP3 [35,36], and we showed that the inhibition of HIF-1α impairs IGF-1-mediated BNIP3 induction [12]. The silencing of Nrf2 has also been linked to impaired HIF-1α activity in breast cancer [37], suggesting that Nrf2 may regulate HIF-1α expression and/or function. In support of this, we observed that while IGF-1 induced HIF-1α in MCF-7 cells transfected with a control siRNA, this induction was significantly impaired when Nrf2 was suppressed (Figure 3A). A similar effect of Nrf2 suppression was observed with DU145 cells, where although the HIF-1α induction in response to IGF-1 was three-fold, this did not reach statistical significance (Figure 3A).

To further investigate the transcriptional regulation of BNIP3 expression, we analyzed the BNIP3 promoter for putative sites of regulation by Nrf2. Applying the ORegAnno tool in the UCSC genome browser, we identified HIF-1α binding sites, nuclear respiratory factor 1 (NRF1) binding sites and a known Nrf2-inducible target gene [38,39], but no Nrf2 binding sites. Other putative transcription factor binding sites in the immediate promoter region included transcription factor AP-2γ (TFAP2C), hepatocyte nuclear factor 4α (HNF4A), ETS proto-oncogene 1, transcription factor (ETS1), E2F transcription factor 4 (E2F4) and melanocyte inducing transcription factor (MITF), of which ETS1 has been reported to be Nrf2-responsive [40]. Using JASPAR2020 to analyze the BNIP3 promoter region 1000 nt upstream of the transcription start site, we identified one potential Nrf2 binding site with a relative score above 0.8, which could facilitate direct activation of BNIP3 transcription through Nrf2. However, by applying a more stringent threshold of 0.9 for the relative score, we identified three binding sites for HIF-1α, and seven binding sites for NRF1, suggesting that these may be important sites (Supplementary Materials Figure S2A,B). Due to its well-established association with mitochondrial biogenesis [41,42], and the observation of multiple putative binding sites in the promoter, we tested whether NRF1 is a component of the Nrf2 pathway for the induction of BNIP3. To test this, NRF1 was suppressed using siRNA in MCF-7 cells and the levels were assessed using RT-qPCR (Figure 3D). NRF1 suppression resulted in impaired induction of BNIP3 by IGF-1 compared to control cells (Figure 3C). Of note, the suppression of NRF1 and any resulting reduction in mitochondrial biogenesis did not decrease mitochondrial mass, as indicated by the unchanged levels of the mitochondrial markers PHB1 and TOM20 (Figure 3E). Therefore, the observed reduction in BNIP3 was likely due to impaired induction rather than reduced mitochondrial mass. Overall, the data indicate that Nrf2 could regulate BNIP3 expression through either NRF1 or HIF-1α. Moreover, these data suggest a role for NRF1 in BNIP3 function, as well as in mitochondrial biogenesis.

### 3.4. The IGF-1-Nrf2-BNIP3 Pathway Regulates Mitochondrial Dynamics and Cellular Capacity for Autophagosomal Turnover in Response to Metabolic and Mitochondrial Stress

**Figure 4:**
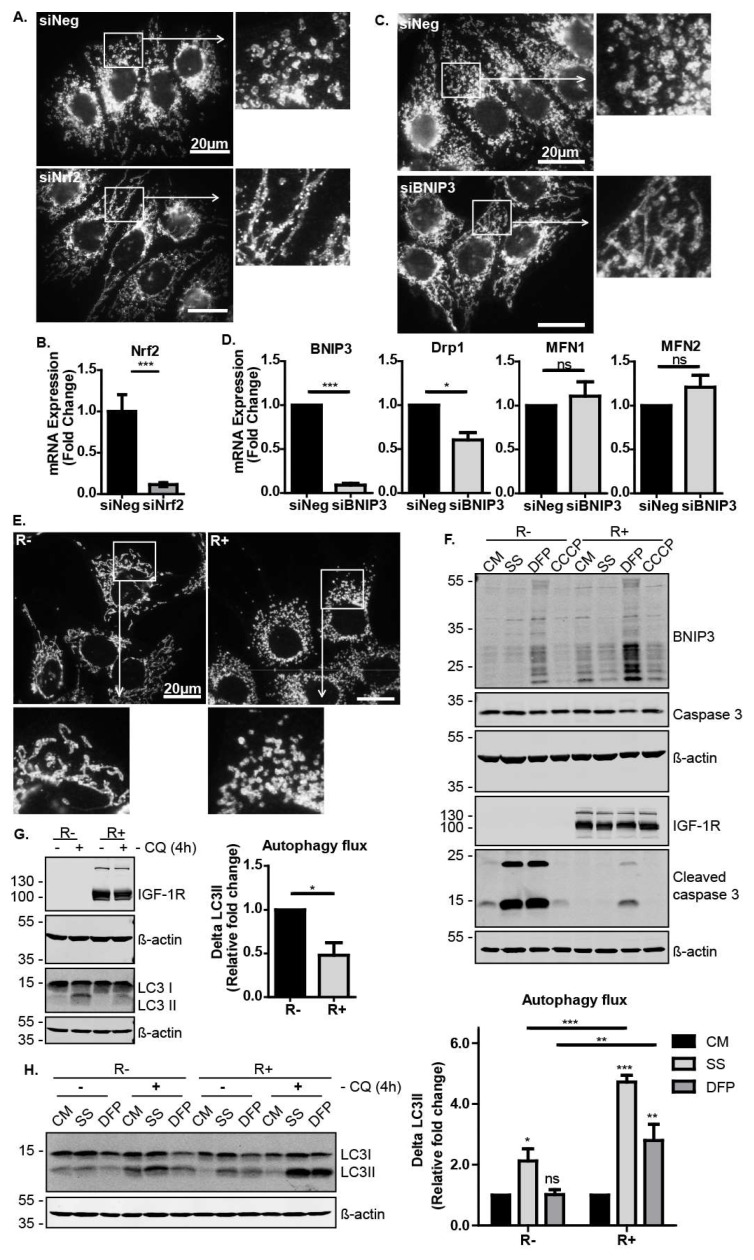
The IGF-1-Nrf2-BNIP3 pathway regulates mitochondrial morphology and turnover. (**A**) Immunofluorescence showing TOM20 in MCF-7 cells transfected with siNeg or siNrf2 cultured in CM. Cells were stained with anti-TOM20 antibody at 72 h post transfection. Images were captured at 1000× magnification, and the enlarged images are 3× zooms. (**B**) Gene expression levels of Nrf2 in response to transfection with siNeg or siNrf2 in MCF-7 cells were measured using RT-qPCR. (**C**) Immunofluorescence showing TOM20 in MCF-7 cells transfected with siNeg or siBNIP3 cultured in the CM at 72 h post transfection. Images were captured at 1000× magnification, and the enlarged images are 3× zooms. (**D**) Gene expression levels of BNIP3 and the mitochondria dynamics regulatory genes Drp1, MFN1 and MFN2 measured using RT-qPCR in MCF-7 cells transfected with siNeg or siBNIP3 for 72 h. (**E**) Immunofluorescence showing TOM20 in R− and R+ cells cultured in the CM. Images were captured at 1000× magnification, and the enlarged images are 3× zooms. (**F**) Western blots showing BNIP3 and cleaved caspase 3 levels after 24 h serum starvation or in the CM in the absence or presence of DFP (1 mM) or CCCP (10 μM). (**G**) Western blots showing LC3 levels in R− and R+ cells. Cells were cultured in CM in the absence or presence of chloroquine (50 μM) added 4 h before harvesting. The autophagic flux was calculated as ΔLC3II for each condition in the presence and absence of chloroquine and presented as a fold change relative to the R− sample. (**H**) Western blot showing LC3 levels in R− and R+ cells cultured in CM with or without DFP (1 mM) or in response to serum starvation for 24 h. Chloroquine (50 μM) was added to the indicated cultures for the last 4 h before harvest. In all figures, the data presented was derived from three independent experiments. For Western blots, protein levels were normalised to β-actin and presented as a fold change relative to the control sample set to a value of 1. For RT-qPCR, gene expression levels were normalised to the housekeeping gene UBC and presented as a fold change relative to control conditions set at a value of 1. Statistical analysis was performed using one-way ANOVA (**A**,**B**) and the Student’s *t*-test (**C**) (*: *p* < 0.05, **: *p* < 0.01, ***: *p* < 0.001). CQ: chloroquine.

We next explored Nrf2 activity in mitochondrial dynamics and mitophagy/autophagy. We first observed that Nrf2 suppression in MCF-7 cells had a robust effect on the mitochondrial morphology, as shown by immunofluorescence with the mitochondrial marker TOM20 (Figure 4A,B). Cells in which Nrf2 was suppressed exhibited more fused and elongated mitochondria than the control cells (Figure 4A). Similarly, the suppression of BNIP3 resulted in mitochondrial elongation (Figure 4C). Since the accumulation of elongated mitochondria could be due to reduced mitochondrial fission or enhanced mitochondrial fusion, we analysed the expression levels of known regulators of mitochondrial dynamics. We observed that BNIP3 suppression resulted in lower levels of Drp1 (Figure 4D), which is a mitochondria fission mediator known to be recruited by BNIP3 [43]. This indicates that these cells have reduced mitochondrial fission, which generally precedes and facilitates mitophagy [44]. Thus, the IGF-1–Nrf2–BNIP3 signalling pathway is required for regulation of mitochondrial morphology and dynamics, potentially affecting cell capacity to induce mitochondrial clearance. Interestingly, we also found that the R− cells exhibited more elongated mitochondria than R+ cells, again indicating a requirement for IGF-1 signals in mitochondrial dynamics (Figure 4E).

Next, we sought to investigate the physiological relevance of IGF-1 signalling in mitophagy induction during cellular stress responses. To test this, R− and R+ cells were exposed to serum starvation to induce metabolic stress, or to either the iron chelator deferiprone (DFP) or the mitochondrial uncoupler carbonyl cyanide m-chlorophenyl hydrazine (CCCP) to induce mitochondrial stress. Interestingly, DFP induced BNIP3 expression in both cell lines, suggesting that DFP induces mitophagy via BNIP3. However, DFP-induced BNIP3 expression was clearly more evident in R+ cells than in R− cells. Furthermore, R− cells exhibited a significantly lower tolerance to serum starvation and DFP than R+ cells and accumulated higher levels of caspase 3 cleavage indicative of apoptosis (Figure 4F). These results show that a loss of IGF-1R signalling resulted in impaired cellular stress responses leading to cell death.

We next investigated the levels of autophagosomal turnover (including mitophagy) by measuring autophagic flux in R− and R+ cells exposed to stress. Autophagic flux is derived by measuring protein levels of LC3II and calculating the difference in levels (ΔLC3II) for each culture condition in the presence and absence of the lysosomal inhibitor chloroquine (CQ). As can be seen in Figure 4G, R− cells displayed higher levels of autophagosomal turnover than R+ cells in control cultures. However, when the cells were exposed to serum starvation or DFP, R+ cells exhibited a higher capacity to induce autophagosome turnover, suggesting that IGF-1 signalling promoted both general autophagy and mitophagy in response to stress (Figure 4H).

Taken together, these data indicate that IGF-1 signalling supports cell survival in response to autophagic and mitophagic stresses, and that this is mediated by increasing the capacity of the cell to induce autophagosome turnover. The IGF-1-mediated induction of BNIP3 in a Nrf2-dependent manner may, thus, be a component of an overall cell survival and stress protective response.

## 4. Discussion

IGF-1 signalling is essential for growth and promotes tumourigenesis, cancer cell survival and proliferation, as well as having a highly conserved and potent effect on mitochondrial function and homeostasis [1,2,9]. The loss of IGF-1 signals leading to impaired mitochondrial capacity for ATP production and antioxidant capacity are effects that can be reversed by IGF-1 therapy, suggesting that IGF-1 signalling is mitochondria protective [9].

In this study, we established that IGF-1 mediates a conserved signal through Nrf2 for the induction of BNIP3 expression in cancer cells and MEFs. Nrf2 has a well-described cytoprotective function in its antioxidant stress response and detoxification. In cancer cells, this could be exploited to allow cells to better survive in stressful environments and facilitate the development of chemoresistance [45,46]. We observed that the IGF-1-mediated induction of BNIP3 [12] can occur through either HIF-1α or NRF1, both of which are induced by Nrf2 [37,39,47], suggesting that BNIP3 may be a secondary, rather than a direct, Nrf2 target gene. The observation that NRF1 may mediate the induction of BNIP3 is interesting because it is better known to induce mitochondrial biogenesis rather than mitophagy [42]. We previously showed that IGF-1 induces NRF1 in the context of mitochondrial biogenesis [12]. However, our observations here of NRF1 acting downstream of IGF-1 and Nrf2 support the conclusion that the synthesis and turnover of mitochondria are coupled through an IGF-1–Nrf2–BNIP3 pathway.

Mitophagy is important for the overall maintenance of cellular health, and mitophagy dysregulation is common in many diseases [48]. Mitophagy is also central to cell differentiation, which requires careful regulation of mitochondrial mass and distribution to ensure that the mitochondrial function of the cell matches the specific cellular requirements for, e.g., energy production and reactive oxygen species (ROS) clearance [49,50]. BNIP3 was found to enhance mitochondrial function under hypoxic conditions in stem cells and to facilitate differentiation of epidermal keratinocytes [51,52]. However, this central role of mitophagy in the differentiation of various cell types enables mitophagic processes to induce de-differentiation of otherwise fully differentiated cells in cancer progression, allowing cancer cells to acquire stemness features [53]. Interestingly, the IGF-1 signalling pathway has been proposed to maintain both stem-like qualities and to enhance cell survival during cell differentiation [54,55].

Our findings suggest that IGF-1 signals are essential for the maintenance of mitochondrial homeostasis. The suppression of either Nrf2 or BNIP3 in MCF-7 cells caused a dramatically altered mitochondrial appearance with more fused and elongated mitochondria, a morphological difference that was clearly more evident in R− cells than R+ cells. The suppression of BNIP3 was also associated with reduced expression of the mitochondrial fission mediator Drp1, which is known to be recruited by BNIP3 to facilitate mitophagy [43]. Thus, IGF-induced BNIP3 can regulate mitochondrial dynamics. Interestingly, the punctate mitochondria and higher levels of Drp1 observed in control MCF-7 cells relative to cells with suppressed Nrf2 or BNIP3 have also been described as characteristics of stem cells [49].

BNIP3 is a protein with at least a dual function having the ability to promote cell death under certain conditions and to induce mitophagy, particularly under hypoxic conditions [56]. It has also been implicated in regulating cell proliferation [57]. Since IGF-1 induces BNIP3 in serum-deprived cells, while IGF-1 is known to protect against apoptosis, it is likely that BNIP3 induces mitophagy in these conditions. This conclusion is supported by our observations on autophagic flux in R− and R+ cells. Higher basal levels of autophagic flux were present in R− cells compared to R+ cells, as could be expected due to the activating effects of IGF-1 on mTORC1, leading to inhibition of general autophagy [58]. However, R+ cells induced autophagosomal turnover in response to either metabolic or mitochondrial stress significantly more potently than R− cells, suggesting that IGF-1 signalling increased the capacity of cells to induce both autophagy and mitophagy. We also observed a striking difference between R− and R+ cells in terms of their sensitivity to cellular stress induced by either serum starvation or DFP. R+ cells clearly displayed lower levels of apoptosis under these conditions than R− cells, suggesting that the IGF-1 pathway increased the cellular tolerance to such stresses. The elevated levels of BNIP3 in R+ cells would be expected to mediate efficient mitophagic clearance in response to DFP. Furthermore, BNIP3 has previously been shown to enhance cell survival in conditions of nutrient deprivation [59], which may contribute to the increased survival observed here with R+ cells in serum-deprived cultures. It is also interesting that neither R− nor R+ cells exhibited cell death in response to CCCP, which is known to upregulate mitophagy through the PINK1-Parkin-mediated pathway [60,61], despite an observed CCCP induction of PINK1 (see Supplementary Materials Figure S1F). This suggests that R− cells that lack IGF-1 signaling may be uniquely impaired in BNIP3-mediated mitophagy. Therefore, although alternative functions of BNIP3 cannot yet be ruled out, we propose that BNIP3 expression levels are central to the dramatic differences observed in autophagic flux and cell survival between R− and R+ cells in response to either serum starvation or DFP.

In conclusion, our study demonstrates that IGF-1 signalling has an essential function in mitochondrial dynamics and turnover through the activity of Nrf2 and BNIP3. This mitochondria-protective role may be a key vulnerability of this essential growth pathway and offer potential for novel therapeutic intervention in cancer.

## Supplementary Materials

The following are available online at https://www.mdpi.com/2073-4409/9/1/147/s1.
