# Supplementary material for: IGF-1 Signalling Regulates Mitochondria Dynamics and Turnover through a Conserved GSK-3β–Nrf2–BNIP3 Pathway
Source: Cells. 2020 Jan 8;9(1):147. doi: 10.3390/cells9010147 (PMC7016769; doi:10.3390/cells9010147)
Supplement: Supplementary file 1 [file cells-09-00147-s001.pdf]

Supplementary Table 1.

Oligonucleotide primer sequences used for RQ-PCR.

Gene:		Primer sequence:	Source:
BNIP3	F:	5’-CGTTCCAGCCTCGGTTTCTA-3’	Recognizes: NM_004052.3 yielding a product of 133nt.
	R:	5’-ATCTTGTGGTGTCTGCGAGC-3’	
Drp1	F:	5'-TGAAGGATGTCATGTCGGACC-3'	WAN, Y. Y.et al. 2014. Involvement of Drp1 in hypoxia-induced migration of human glioblastoma U251 cells. Oncol Rep, 32, 619-26.
	R:	5'-GTTGAGGACGTTGACTTGGCT-3'	
GCLC	F:	5'-GGCACAAGGACGTTCTCAAGT-3'	JIANG, M., et al. 2015. BMP-driven NRF2 activation in esophageal basal cell differentiation and eosinophilic esophagitis. The Journal of Clinical Investigation, 125, 1557-1568.
	R:	5'-CAGACAGGACCAACCGGAC-3'	
HO1	F:	5'-CTCAAACCTCCAAAAGCC-3'	ZHONG, Z. & TANG, Y. 2016. Upregulation of Periostin Prevents High Glucose-Induced Mitochondrial Apoptosis in Human Umbilical Vein Endothelial Cells via Activation of Nrf2/HO-1 Signaling. Cellular Physiology and Biochemistry, 39, 71-80.
	R:	5'-TCAAAAACCACCCCAACCC-3'	
KEAP1	F:	5'-TTCAAGGCCATGTTCACCAA-3'	DEVLING, T. W. P. et al. 2005. Utility of siRNA against Keap1 as a strategy to stimulate a cancer chemopreventive phenotype. Proceedings of the National Academy of Sciences of the United States of America, 102, 7280-7285A.
	R:	5'-TGGATACCCTCAATGGACACC-3'	
MFN1	F:	5’-TGTTTTGGTCGCAAACTCTG-3’	RUSSELL, A. P. et al. 2013. Regulation of miRNAs in human skeletal muscle following acute endurance exercise and short-term endurance training. The Journal of physiology, 591, 4637-4653.
	R:	5’-CTGTCTGCGTACGTCTTCCA-3’	
MFN2	F:	5'-ATGCATCCCCACTTAAGCAC-3'	RUSSELL, A. P. et al. 2013. Regulation of miRNAs in human skeletal muscle following acute endurance exercise and short-term endurance training. The Journal of physiology, 591, 4637-4653.
	R:	5'-CCAGAGGGCAGAACTTTGTC-3'	
NRF1	F:	5'-CGCTCTGAGAACTTCATGGAGGAACAC-3'	BORI, Z. et al. 2012. The effects of aging, physical training, and a single bout of exercise on mitochondrial protein expression in human skeletal muscle. Experimental gerontology, 47, 417-424.
	R:	5'-GCCACATGGACCTGCTGCACTT-3'	
UBC	F:	5'-TCGAGAATGTCAAGGCAAAGATC-3'	OHL, F. et al. 2005. Gene expression studies in prostate cancer tissue: which reference gene should be selected for normalization? Journal of molecular medicine (Berlin, Germany), 83,<br>1014-1024.
	R:	5'-GAGTGGACTCTTTCTGGATGTTGTA-3'	
Ms/hu Nrf2	F:	5'-CTACTCCCAGGTTGCCCACA-3'	SARR, D. et al. 2017. Oxidative Stress: A Potential Therapeutic Target in Placental Malaria. ImmunoHorizons, 1, 29-41.
	R:	5'-CGACTCATGGTCATCTACAAATGG-3'	
Ms actin	F:	5'-ATATCGCTGCGCTGGTCGTC-3'	Recognizes: NM_007393.5 yielding a product of 149nt.
	R:	5'-ATAGGAGTCCTTCTGACCCATT-3'	
Ms BNIP3	F:	5'-GCTCCCAGACACCACAAGAT-3'	CHAVEZ-VALDEZ, R. et al. 2012. Necrostatin-1 attenuates mitochondrial dysfunction in neurons and astrocytes following neonatal hypoxia-ischemia. Neuroscience, 219, 192-203.
	R:	5'-TGAGAGTAGCTGTGCGCTTC-3'	

List of all primer sequences used for RQ-PCR with human or mouse cell lines. Mouse specific primers sequences are indicated by Ms and the source reference for each primer is included.

Supplementary Figure 1.

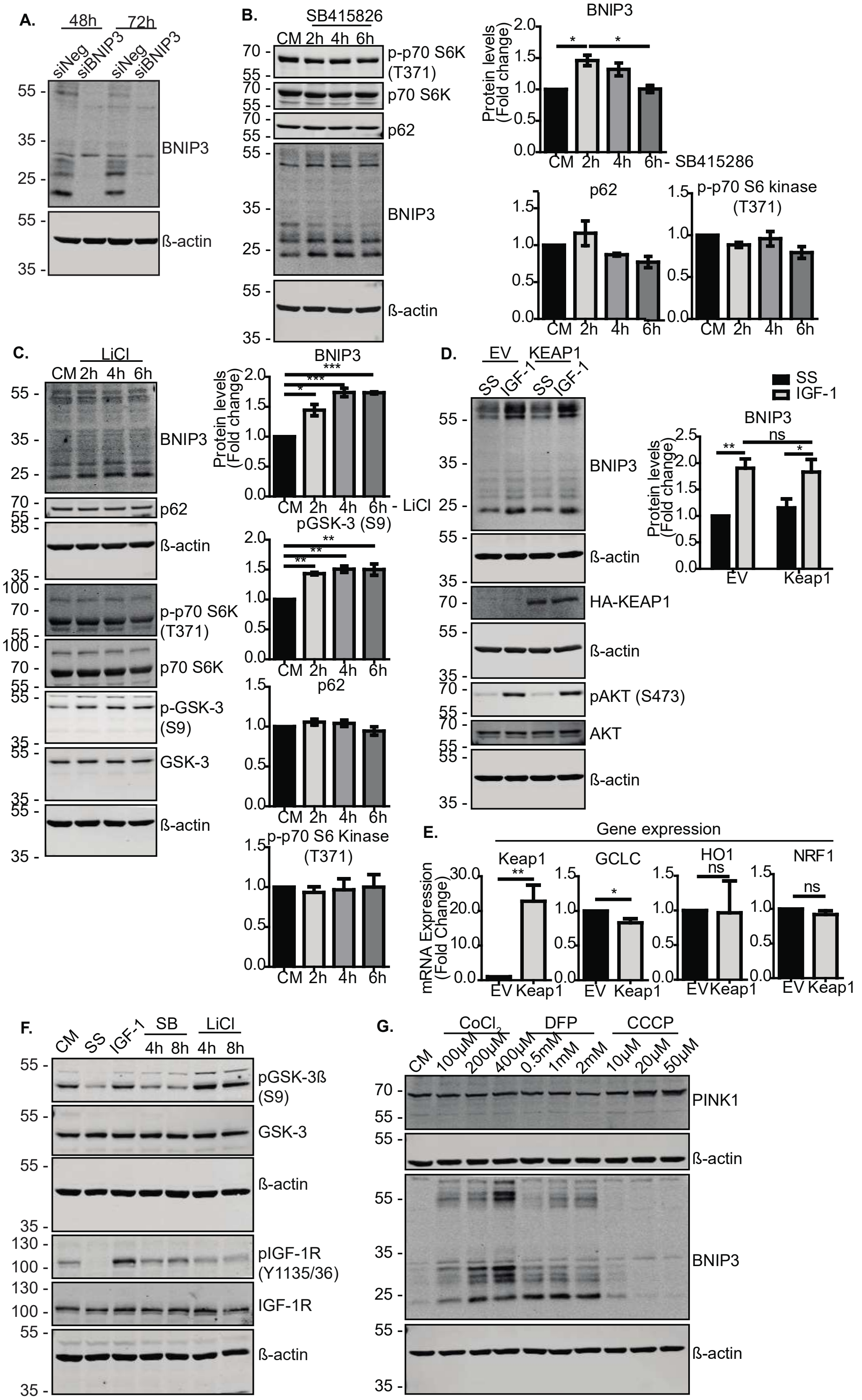

Supplementary Figure 1:

A. Western blot showing BNIP3 levels in MCF-7 cells.

The cells were transfected with negative control siRNA or siRNA targeting BNIP3 for 48 or 72h as indicated on the blot and kept in CM.

***B.* Western blot showing levels of BNIP3, p62 and p-p70 S6 kinase (T371) in MCF-7 cells.**

The cells were kept in control media in the presence or absence of 30μM SB415286 for 2, 4 or 6h.

***C.* Western blot showing levels of BNIP3, pGSK-3β (S9), p62 and p-p70 S6 kinase in DU145 cells.** The cells were maintained in control media in the absence or presence of 10mM LiCl for 2, 4 or 6h.

***D.* Western blot showing DU145 cells transfected with pcDNA3 empty vector (EV) or pcDNA3-HA2-KEAP1.** The cells were serum-starved for 4h prior to addition of IGF-1 (10ng/ml) for 20h.

***E.* mRNA expression levels of KEAP1 and Nrf2 target genes,** GCLC, HO1 and NRF1 in MCF-7 cells transfected with pcDNA3 EV or pcDNA3-HA2-KEAP1 for 48h and maintained in control medium. Levels were measured by RQ-PCR.

***F.* Western blot showing the effects of SB415286 (30μM) and LiCl (10mM) on the GSK-3 status in DU145 cells.** The cells were maintained in CM, serum starved for 4h prior to 4h IGF-1 stimulation (10ng/ml) or had SB415286 or LiCl added to CM for the indicated times.

***G.* Western blot showing levels of PINK1 and BNIP3 in response to $CoCl_2$, DFP or CCCP at the indicated concentrations in DU145 cells.** All exposures were for 24h. In all figures, data is presented from three independent experiments. For Western Blots, protein levels were normalised to β-actin and presented as fold change relative to the control sample set to a value of 1. For RQ-PCR, gene expression levels were normalised to the housekeeping gene UBC and presented as fold change relative to control conditions set at a value of 1.Statistical analysis was performed using one-way ANOVA (Fig. B and C), two-way ANOVA (Fig. D) and Student's t test (Fig. E) (*: $p<0.05$, **: $p<0.01$, ***: $p<0.001$).

Supplemental Figure 2A

Promoter sequence of NFE2L2 gene with potential binding sites for -HIF1α and NRF-1 highlighted.

```
-1000  +ve    TCTTCTTTCTGAAAACTAGTTTTCGCCCTTTACTCTTTCGCTGAAAGGAGACTTTTCACG   -941
-1000  -ve    AGAAGAAAGACTTTTGATCAAAAGCGGGAAATGAGAAAGCGACTTTCCTCTGAAAAGTGC   -941

-940          GAGGGTCAGGGCTCCGACGCTCCGGGGGTGCGGTCCGACCGAGGGTGCCTTCGGCCCGTG   -881
-940          CTCCCAGTCCCGAGGCTGCGAGGCCCCCACGCCAGGCTGGCTCCCACGGAAGCCGGGCAC   -881

-880          GGTGGGCCGGGCTGGTTCGCGGTGAGGCGGGGCACCTGCCCCGCAGGGTGGGGCCCCTGC   -821
-880          CCACCCGGCCCGACCAAGCGCCACTCCGCCCCGTGGACGGGGCGTCCCACCCCGGGGACG   -821

-820          GGGCGGGGTGTGGCGCAAACGTGGGGCCTCCGGGGAACGGCGTCTCCGCCTGCCGCGCGG   -761
-820          CCCGCCCCACACCGCGTTTGCACCCCGGAGGCCCCTTGCCGCAGAGGCGGACGGCGCGCC   -761

-760          AGAGGGCCCGGGGACCCCAGGCGCGGAGGGAGCCCGTCTGAGAAAGCTGAGACGAGCTCG   -701
-760          TCTCCCGGGCCCCTGGGGTCCGCGCCTCCCTCGGGCAGACTCTTTCGACTCTGCTCGAGC   -701

-700          GAGGCGAAGAAGGACGCCCGCCTGCGGGGCCTGTGTTGCCCGGGGCGACAAGTGCGTCCC   -641
-700          CTCCGCTTCTTCCTGCGGGCGGACGCCCCGGACACAACGGGCCCCGCTGTTCACGCAGGG   -641

-640          CGCGGGGCCGCCCCGCCCGTTTCTGGGCCCCTGCGCCAGGGCAGGGCTCTGCGAGTCGAG   -581
-640          GCGCCCCGGCGGGGCGGGCAAAGACCCGGGGACGCGGTCCCGTCCCGAGACGCTCAGCTC   -581

-580          GCCGGGTGGCGAGCGTCGAGGGCGGGGCCCGCGTCCAGGGCTGGGGTGCCCGGCAGAGCC   -521
-580          CGGCCCACCGCTCGCAGCTCCCGCCCCGGGCGCAGGTCCCGACCCCACGGGCCGTCTCGG   -521

-520          TCGGCGTCGCCGGCGAAGGGACGTGCAGGAGTGCGGGGGGCGTGCCTGCGGCGGTCGGGG   -461
-520          AGCCGCAGCGGCCGCTTCCCTGCACGTCCTCACGCCCCCCGCACGGACGCCGCCAGCCCC   -461

-460          CGCGGAGTCAAAGGGGTGATCGTCCTACCTTTCTGCCCGGGGCGGGGCTTCGCATCGCCG   -401
-460          GCGCCTCAGTTTCCCCACTAGCAGGATGGAAAGACGGGCCCCGCCCCGAAGCGTAGCGGC   -401

-400          CAGAGGCACCATCGGTCACGGGTCTCTCAGGCGGCCAGGGTGGCGGGGAAGTTTCCTCTT   -341
-400          GTCTCCGTGGTAGCCAGTGCCCAGAGAGTCCGCCGGTCCCACCGCCCCTTCAAAGGAGAA   -341

-340          GGGCCGGGTGGCGGGCGGCGCCGCCGCTGGCGCGTCGGGTGAGCAGTGCGCCGGGCGCCG   -281
-340          CCCGGCCCACCGCCCGCCGCGGCGGCGACCGCGCAGCCCACTCGTCACGCGGCCCGCGGC   -281

-280          CAGGTCGGGCCCGGCCGAGTGGAGTCCGCCAGCGACGGCGGGAGCGCGGACGCGCGGGGA   -221
-280          GTCCAGCCCGGGCCGGCTCACCTCAGGCGGTCGCTGCCGCCCTCGCGCCTGCGCGCCCCT   -221

-220          GCGGGGCGGGGAGAGGGGCGGGCGCAGGGCGCGTGGCGTCCGGAGACGGGGAGCGGGTGG   -161
-220          CGCCCCGCCCCTCTCCCCGCCCGCGTCCCGCGCACCGCAGGCCTCTGCCCCTCGCCCACC   -161

-160          CGTCCTGGGCGGGGCGCGTGCGCGGCGTGCACGGTGTGCGTGGGGTGCGGGGACGCGTGC   -101
-160          GCAGGACCCGCCCCGCGCACGCGCCGCACGTGCCACACGCACCCCACGCCCCTGCGCACG   -101

-100          GCGTCCGGGGTTCAGCGCCGGTTACCCGCTGCGCCGGCGTCTAGGCGGGCCGGGGCGGGA   -41
-100          CGCAGGCCCCAAGTCGCGGCCAATGGGCGACGCGGCCGCAGATCCGCCCGGCCCCGCCCT   -41

-40           CGGGACACTCAAGGAGGCCGGCCCGACGCCCCGAGGCGAGTCAGGCCCTCGCGTCGACCC   19
-40           GCCCTGTGAGTTCCTCCGGCCGGGCTGCGGGGCTCCGCTCAGTCCGGGAGCGCAGCTGGG   19

+20           GGCGCCGCGAGGCTGGAGGCGAAAGGGTGGCGGGCGTCGACTTCGTGTAGGGCGTCGGGC   79
+20           CCGCGGCGCTCCGACCTCCGCTTTCCCACCGCCCGCAGCTGAAGCACATCCCGCAGCCCG   79

+80           CGCGCCTGAGGCTAGCGGCGT       +ve    +100
+80           GCGCGGACTCCGATCGCCGCA       -ve    +100
```

The NFE2L2 sequence from 1000 nucleotides before transcriptional start site (-1000) to 100 nucleotides after transcriptional start site (+100) was identified using the Eukaryotic Promoter Database, then overlaid on the BNIP3 sequence in the UCSC Genome Browser, and analysed with the OReGanno tool for potential transcription factor binding sites. The JASPAR scan analysis function was used to predict the binding of NRF1 (matrix ID MA0506.1), HIF-1 α (MA1106.1) and Nrf2 (MA0150.1). NFE2L2 binding sites are highlighted in red, HIF1α binding sites are highlighted in blue and NRF1 binding sites are highlighted in green.

Supplemental Figure 2B

List of putative binding sites for HIF1α, NRF-1 and NFE2L2 in BNIP3 promoter with assigned scores relative to the consensus binding sites.

Name	Score	Relative score	Start	End	Strand	Predicted sequence
**NRF1**	17.5512	0.993742	-235	-225	-	GCGCCTGCGCG
**NRF1**	17.0771	0.987886	-107	-97	+	GCGCGTGCGCA
**NRF1**	16.5703	0.981627	-146	-136	+	GCGCGTGCGCG
**NRF1**	14.6196	0.957536	-106	-96	-	GCGCACGCGCA
**NRF1**	14.4887	0.955919	-236	-226	+	GCGCAGGCGCG
**NRF1**	13.6892	0.946046	-112	-102	-	GCCCCTGCGCA
**NRF1**	13.1574	0.939479	-145	-135	-	GCGCACGCGCC
**HIF1A**	12.3625	0.983205	-136	-127	+	GCACGTGCGG
**HIF1A**	12.017	0.975072	-500	-491	+	GGACGTGCAG
**HIF1A**	11.8335	0.97075	-134	-125	-	GCACGTGCCA
**NFE2L2**	8.92847	0.823625	-969	-959	-	ATGAGAAAGCG

Transcription factors in the BNIP3 promoter sequence from Eukaryotic Promoter Database. The JASPAR scan analysis software generates a score and a relative score based on the similarity between the probed sequence and the transcription factor consensus sequence. These scores and the relevant consensus sequences are indicated in the list.
